# An unusual foreign body (sewing needle tip) in the tonsils

**DOI:** 10.1016/S1808-8694(15)30558-9

**Published:** 2015-10-19

**Authors:** Shitij Arora, J.K. Sharma, S.K. Pippal, Yatin Sethi, Abhinav Yadav, Swapnil Brajpuria

**Affiliations:** 1 DR (resident ENT, gandhi medial college bhopal india); 2 DR. (prof & head, dep.of ENT, gandhi medial college bhopal india); 3 DR. (assos. prof, dep of ENT, gandhi medial college bhopal india); 4 DR. resident ENT, gandhi medial college bhopal india; 5 DR. resident ENT, gandhi medial college bhopal india; 6 DR. resident ENT, gandhi medial college bhopal india

**Keywords:** tonsil, foreign body, sewing needle tip

## INTRODUCTION

Foreign bodies in the throat are common emergencies in ear nose and throat practice. Usual foreign bodies includes coin, bone piece, fish bone, nails, button, glass pieces, denture, ear ring, chain, pins and needles^1^,^2^,^3^.

There are very few reported cases of a foreign body in the tonsillar substance. Pharyngeal foreign bodies get lodged in the mucosa and may not be visible on routine ENT examination.

It is always mandatory to give due respect and care to the complaints of the patient and do a proper ear, nose and throat examination, get the investigations done as needed so as not to under diagnose the case or leave a foreign body and give constant irritation and misery to the patient^4^.

## CASE REPORT

A 30 yr old female presented at the Department of ENT Hamidia Hospital & Gandhi Medical College, Bhopal with complaint of foreign body sensation and pain in throat for past one month. Patient gave history of accidental ingestion of a part of sewing needle while sewing cloth one month back.

Examination of the oropharynx was done. Tonsils on both side were enlarged 3+ and congested. Anterior pillar was flushed. No other abnormality was detected. Tonsils were palpated and no metallic object was palpated.

Indirect laryngoscopic examination was normal. X-ray soft tissue neck AP and Lateral views were done but no foreign body was visualised. Direct laryngoscopy was done under general anaesthesia. All structures seen normal and no foreign body visualised.

**Figure 1:**
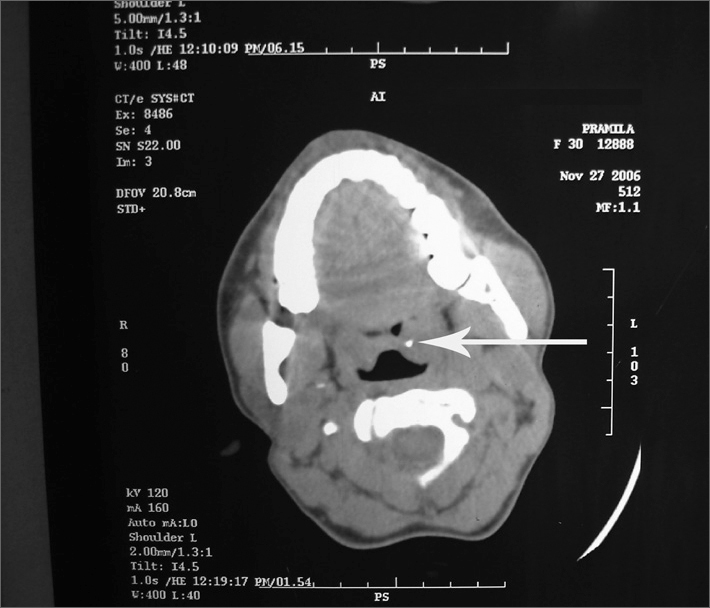
CT scan showing a small metallic density in left tonsil (arrow)

CT SCAN of neck (p) was done. Serial axial slices of 5mm were taken and it showed a small metallic density of +400 to +450 HU of size 3×3×3 mm involving the substance of the Left tonsil. Visualised nasopharyngeal, oropharyngeal and hypopharyngeal air spaces were normal. (fig 1)

Tonsillectomy was done under general anaesthesia. Left tonsil was cut and a 3mm metallic pin seen with in the tonsil tissue. The outcome of the patient was excellent. No complications were seen after a 3 month follow up.

## DISCUSSION

Foreign bodies in throat are common emergencies in ENT practice. Forgotten foreign body become troublesome in presence of complications and require a thorough investigation of any case of dysphagia with odynophagia^4^.

Usually they pass harmlessly through the gastrointestinal tract but a few become impacted at various levels of pharyngeal soft tissue^5^. History of ingestion of foreign body, inability to swallow saliva and dysphagia are most important diagnostic criteria^1^.

If the impacted foreign body is radioluscent, in the presence of positive history, symptoms or clinical suspicion, endoscopic examination is suggested. The diagnosis of radio-opaque foreign body ingestion does not pose a major problem. However, it is crucial to take a radiograph from the pharynx, where the foreign body is most likely to become impacted to the level of pylorus

In this particular case there was a foreign body sensation in throat with odynophagia with positive history of accidental ingestion foreign body. Plain radiographic and endoscopic evaluation of upper airway is inconclusive with CT scan neck suggestive of a metallic foreign body of 3×3×3 mm embedded completely in substance of left tonsil Tonsillectomy was done and the patient was relieved.
